# Sodium-glucose co-transporter-2 inhibitor (SGLT2i) treatment and risk of osteomyelitis: A pharmacovigilance study of the FAERS database

**DOI:** 10.3389/fphar.2023.1110575

**Published:** 2023-02-14

**Authors:** Hui Zhao, Zi-Ran Li, Qian Zhang, Ming-Kang Zhong, Ming-Ming Yan, Xiao-Yan Qiu

**Affiliations:** Clinical Pharmacy Department, Huashan Hospital, Fudan University, Shanghai, China

**Keywords:** FAERS, osteomyelitis, diabetes mellitus, SGLT2is, canagliflozin, pharmacovigilance

## Abstract

**Background and purpose:** Several clinical trials have indicated that the use of canagliflozin increases the risk of lower extremity amputation. Although the US Food and Drug Administration (FDA) has withdrawn its black box warning about amputation risk for canagliflozin, the risk still exists. We sought to estimate the association between hypoglycemic medications, especially sodium-glucose co-transporter-2 inhibitors (SGLT2is), and adverse events (AEs) before the irreversible outcome of amputation as a promising early warning, based on the FDA Adverse Event Reporting System (FAERS) data.

**Methods:** Publicly available FAERS data were analyzed using a reporting odds ratio (ROR) method and validated by a Bayesian confidence propagation neural network (BCPNN) method. The developing trend of the ROR was investigated by a series of calculations based on the accumulation of data in the FAERS database quarter by quarter.

**Results:** Ketoacidosis, infection, peripheral ischemia, renal impairment, and inflammation including osteomyelitis might be more likely to occur among users of SGLT2is, especially canagliflozin. Osteomyelitis and cellulitis are AEs unique to canagliflozin. Among 2,888 osteomyelitis-related reports referring to hypoglycemic medications, 2,333 cases were associated with SGLT2is, with canagliflozin accounting for 2,283 of these cases and generating an ROR value of 360.89 and a lower limit of information component (IC_025_) of 7.79. No BCPNN-positive signal could be generated for drugs other than insulin and canagliflozin. Reports suggesting that insulin could generate BCPNN-positive signals span from 2004 to 2021, whereas reports with BCPNN-positive signals emerged only since the second quarter (Q2) of 2017, 4 years since the approval of SGLT2is in Q2 of 2013, for canagliflozin and drug groups containing canagliflozin.

**Conclusion:** This data-mining investigation revealed a strong association between canagliflozin treatment and developing osteomyelitis that might be a crucial forewarning to lower extremity amputation. Further studies with updated data are needed to better characterize the risk of osteomyelitis associated with SGLT2is.

## 1 Introduction

Sodium-glucose co-transporter-2 inhibitors (SGLT2is) are a class of oral hypoglycemic agents that exert their glucose-lowering effect by lowering the renal threshold for glucose reabsorption in the proximal renal tubule, causing glycosuria, and increasing renal excretion of glucose. In patients with type 2 diabetes (T2D), SGLT2is are effective in controlling glycemia, blood pressure, and body weight (Zaccardi et al., 2016). Since the approval of canagliflozin in 2013, this drug has been reported to demonstrate a protective effect against renal and cardiovascular disease (CVD) (Perkovic et al., 2019; Zelniker et al., 2019; Bauersachs et al., 2022), thus preventing hospitalization for heart failure (HF) in patients with T2D with or without a prior history of HF or CVD at baseline (Mahaffey et al., 2018), and significantly improving outcomes for patients with HF and reduced ejection fraction, including 42%–50% of patients with T2D (Mahaffey et al., 2019).

However, in 2017, the US Food and Drug Administration (FDA) issued a drug safety communication regarding a boxed warning about foot and leg amputations with the use of canagliflozin and removed it in 2020, reconsidering its additional benefits. The amputation risk with canagliflozin remains and is still described in the warnings and precautions section of the prescribing information. Healthcare professionals and patients should continue to recognize the importance of preventative foot care and monitor for new pain, tenderness, sores, ulcers, and infections in the legs and feet. Risk factors that may predispose patients to the need for amputation should be considered when choosing antidiabetic medicines. This warning is based on evidence from two clinical trials. The Canagliflozin Cardiovascular Assessment Study (CANVAS) program used data from two trials and showed that there was a statistically significantly higher risk of amputation with canagliflozin than with placebo (6.3 *vs*. 3.4 participants with amputations per 1,000 patient-years) (Neal et al., 2015; Fulcher et al., 2016). A retrospective cohort study raised concerns in relation to the increased risk of lower extremity amputation with canagliflozin, and it remains unclear whether and to what extent this side effect could also occur with other SGLT2is, which are also reported to have the risk of osteomyelitis (Chang et al., 2018).

To the best of our knowledge, there is no grand assessment of the association between all classes of hypoglycemic drugs and adverse events (AEs), which might be precursors to lower extremity amputation, especially osteomyelitis, based on real-world data. Osteomyelitis is an inflammatory bone disease that is caused by an infectious microorganism and leads to progressive bone destruction and loss (Prokesch et al., 2016; Kavanagh et al., 2018), which complicates approximately 10%–20% of foot ulcers in individuals with diabetes attending specialist clinics (Shone et al., 2006), although a frequency as high as 68% has been reported in a study (Newman et al., 1991; Schwegler et al., 2008). This complication greatly increases the risk of lower extremity amputation (Lavery et al., 2006; Game, 2010). Although both the CANVAS and Canagliflozin Cardiovascular Assessment Study–Renal (CANVAS-R) trials suggested an increased risk for lower limb amputations, they underestimated the risk of osteomyelitis, since its treatment might greatly reduce the risk of amputation. In this study, based on the US FDA Adverse Event Reporting System (FAERS), we investigated the association between treatment with hypoglycemic drugs and the AEs mentioned, as well as the association between diabetes and AEs. Some drug–AE pairs could generate stronger signals than pairs of the same AEs and diabetes, especially osteonecrosis-related AEs. These stronger signals could be used as a warning for the prognosis of lower extremity amputation, whereas minor signals of drug–AE pairs compared with those of diabetes–AE pairs could be considered to demonstrate curative effects.

## 2 Methods

### 2.1 Data source

**FIGURE 1:**
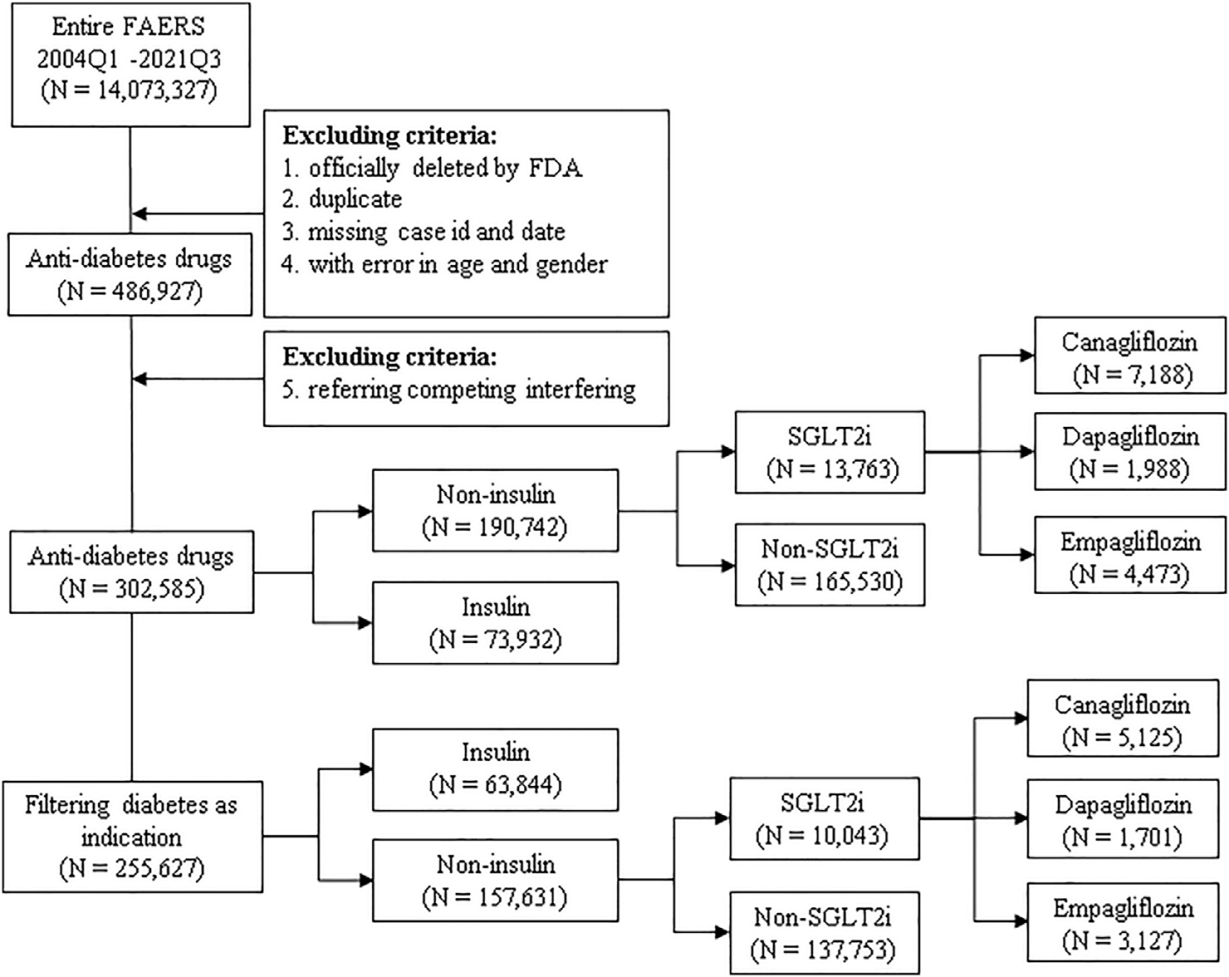
Scheme of the study. Publicly available FAERS data from 1 January 2004 to 30 September 2021 were filtered using the exclusion criteria, and all included reports were categorized into subgroups and analyzed for association with osteomyelitis in duplicate with or without filtering diabetes as an indication. N: number of cases of each drug or drug group; insulin: insulin and its analogs; SGLT2is: sodium-glucose co-transporter-2 inhibitors.

Publicly available FAERS data from 1 January 2004 to 30 September 2021 were downloaded from the FDA website as raw data. Hypoglycemic medications were drugs mapped to the Anatomic Therapeutic Chemical Classification (ATC) as A10 class (antidiabetic drugs, ATCA10), including insulin (including insulin and its analogs discussed in this paper) and SGLT2is, as well as biguanides, dipeptidyl peptidase-4 (DPP4), glinides (GLN), glucagon-like peptide 1 (GLP-1), sulfonylureas (SUs), and thiazolidinediones (TZDs). Osteomyelitis was defined as all of the AEs containing the keyword “osteomyelitis,” which were determined by using the Standardized MedDRA Query (SMQ, version 23.0) terms (Katsuhara and Ikeda, 2021), within “Osteonecrosis (SMQ).” The dataset of “Diabetes” was composed of all reports in FAERS containing the keyword “diabetes.” The following criteria of exclusion (Figure 1) were applied: each potential case was subjected to a data-cleaning procedure to remove reports that were officially deleted by FDA authority, that were duplicated, with missing case ID and date, or with inaccurate data for gender and age. The obtained reports were then filtered with the targeted drug as the primary suspected (PS) drug. All the reports containing hypoglycemic medications other than the targeted medication were removed, to minimize the possibility of interfering effects.

### 2.2 Statistical analyses and signal detection

A data-mining procedure using a reporting odds ratio (ROR) method (Min et al., 2018; Moreland-Head et al., 2021) was introduced to investigate the disproportionality in reporting ratio caused by interested drug–AE pairs compared with a random drug–AE pair, which were then evaluated in tandem with a Bayesian confidence propagation neural network (BCPNN) method (Bate et al., 1998), thereby deducing the association between the target drug and event by a prior possibility. The association between diabetes and AEs was also investigated. Drug–AE pairs that could generate stronger signals than the same AEs paired with diabetes were screened out and demonstrated with a heatmap. Osteomyelitis was picked as the major candidate before lower extremity amputation. Data processing was conducted with RStudio 4.1.2, using a logistic regression model. For ROR, a signal was determined as the count of drug–AE pairs greater than 3, plus the value of the ROR higher than 1, and the lower limit of the 95% confidence interval (95% CI) exceeding 1. For BCPNN, a signal was defined as the value of the lower limit of information component (IC_025_) exceeding 0; specifically, an IC_025_ value between 0 and 1.5 was defined as a weak signal, an IC_025_ value between 1.5 and 3 was considered as a medium signal, and an IC_025_ value > 3 was considered as a strong signal.

### 2.3 Data mining for osteomyelitis-related cases

All aforementioned drugs and drug groups were subjected to descriptive analysis for demographics, including gender, age category, annual report counts, occupation of the reporter, role of the targeted drug, and outcomes. Because hypoglycemic medications may sometimes be used by non-diabetic individuals or for non-diabetic purposes (Zhu et al., 2020; Bonora et al., 2021), many reports present no specific indications or missing information, and all interested drugs or drug groups were analyzed in duplicate with or without filtering diabetes as an indication (Figure 1). Reports referring to competing interfering indications such as from drugs known for causing osteomyelitis, including zoledronic acid and alendronate sodium, were excluded, as well as reports listing osteology conditions as indications and AEs, because osteomyelitis may occur preferentially in patients with diabetic ulcers, lower extremity amputation, and metatarsal excision (Game, 2010). Because osteomyelitis might occur preferentially in patients with known infections (Lavery et al., 2006), we excluded reports containing competing indications and AEs that are typically reported preferentially among users of SGLT2is, to minimize the bias due to dilution or competition (Davies et al., 2018; Pasquel et al., 2021), such as diabetic foot ulcers (Ramsey et al., 1999) and infections (Eckman et al., 1995), especially genital, genitourinary tract, and urinary tract infections, diabetic ketoacidosis, and Fournier’s gangrene, as well as reports listing all antibiotics or becaplermin (Kobayashi et al., 2022). Furthermore, because the use of insulin and its analogs is typically considered a proxy of disease severity or advanced disease stage (Davies et al., 2018; Pasquel et al., 2021), we categorized reports referring to insulin as a control group. In addition**,** the gender bias in the osteomyelitis reports was investigated. Reports referring to testosterone and estrogen were extracted and filtered as described earlier.

The developing trend of RORs on a quarterly basis was investigated. We designed a procedure to mimic the accumulation of FAERS data in real world by adding up every quarter of data into the dataset. A series of quarterly ROR (q-ROR) values was generated for interested drug/drug group–osteomyelitis pairs. Chi-square (Chi2) tests were performed to compare the changing tendencies of q-ROR values of given pairs, as well as the tendencies before and after SGLT2is were approved by the FDA, to eliminate the interfering effect caused by comorbidities or concomitants.

## 3 Results

### 3.1 Heatmap of IC_025_ generated by hypoglycemic medications and diabetes paired with AEs

**FIGURE 2:**
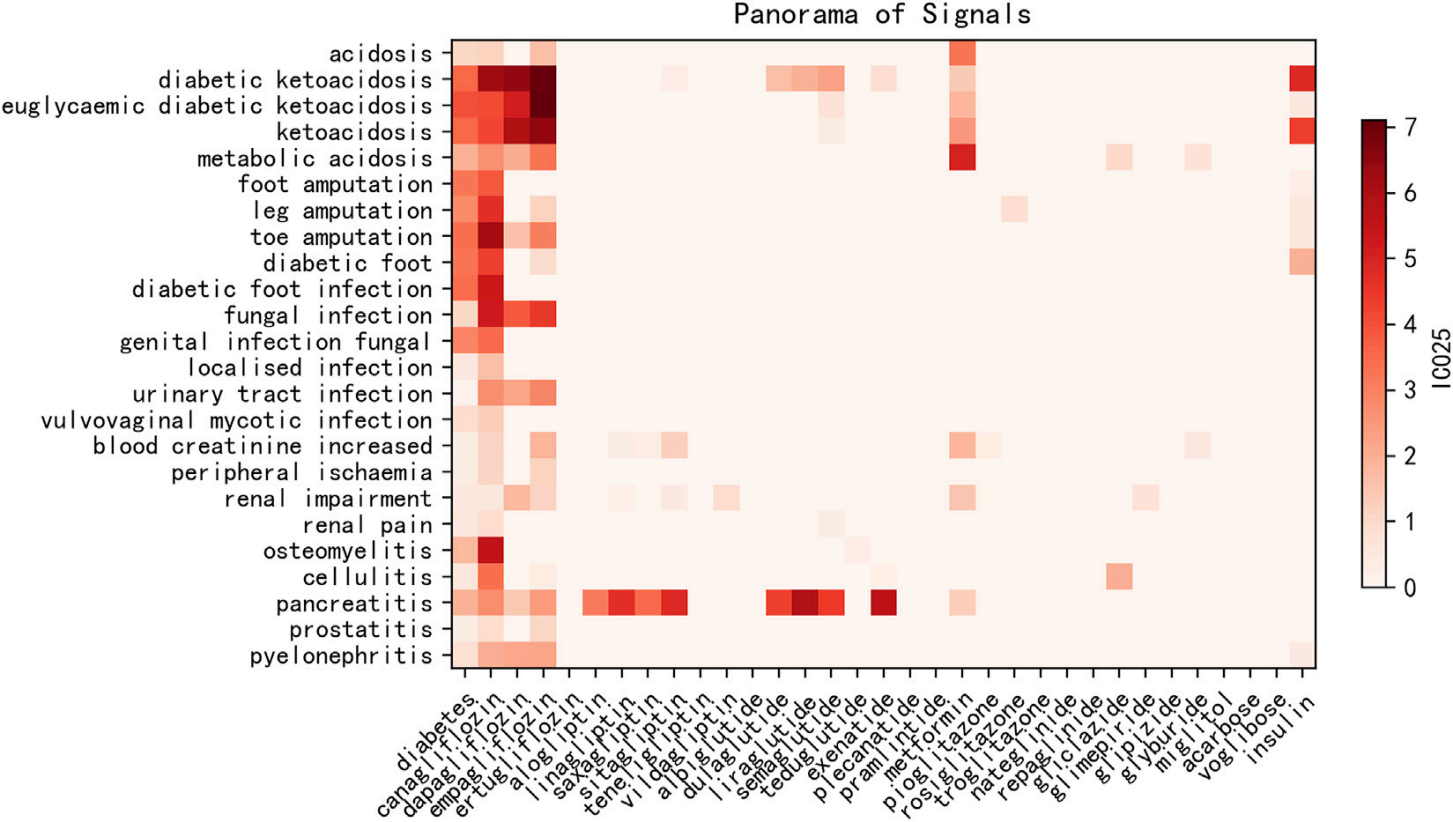
Heatmap of IC_025_ values of blood glucose-lowering drugs and associated risk AEs. *x*-axis: diabetes and blood glucose-lowering drugs; *y*-axis: risk AEs with IC_025_ values higher than that of diabetes–AE pairs. IC_025_: lower limit of the information component.

As shown in Figure 2 and Supplementary Table S1, compared with the risk factor diabetes, most of the hypoglycemic drugs demonstrated curative effects for patients with diabetes, whereas SGLT2is might increase the risk of ketoacidosis (IC_025_ of diabetes: 3.57 *vs*. IC_025_ of empagliflozin: 6.99), lower limb extremity amputation such as toe amputation (IC_025_ of diabetes: 3.38 *vs*. IC_025_ of canagliflozin: 5.35), gangrene such as Fournier’s gangrene (IC_025_ of diabetes: 3.75 *vs*. IC_025_ of empagliflozin: 6.76), infection such as urinary tract infection (IC_025_ of diabetes: 0.10 *vs*. IC_025_ of canagliflozin: 1.84), ulcer such as skin ulcer (IC_025_ of diabetes: 0.79 *vs.* IC_025_ of canagliflozin: 2.42), peripheral ischemia (IC_025_ of diabetes: 0.39 *vs*. IC_025_ of canagliflozin: 2.23), kidney injury such as acute kidney injury (IC_025_ of diabetes: 1.56 *vs*. IC_025_ of dapagliflozin: 2.27), and various inflammations including osteomyelitis, fasciitis, and cellulitis, especially for canagliflozin. Osteomyelitis (IC_025_ of diabetes: 1.80 *vs*. IC_025_ of canagliflozin: 4.17) and cellulitis (IC_025_ of diabetes: 0.56 *vs*. IC_025_ of canagliflozin: 2.16) were AEs unique to canagliflozin.

### 3.2 Demography of osteomyelitis-related cases

The FAERS database is composed of a total of 14,073,327 AE reports from 1 January 2004 to 30 September 2021. After applying the data-cleansing procedure described earlier, there were 2,888 osteomyelitis-related reports referring to hypoglycemic drugs, among which 2,333 reports were associated with SGLT2is, especially canagliflozin (2,283 reports; Table 1). Among reports referring to both canagliflozin and osteomyelitis, 73.50% of patients are male, whereas the gross gender ratio for each category of hypoglycemic drugs is relatively balanced. Among all osteomyelitis-related patients treated with canagliflozin, 23.74% of patients are 18–29 years old, 59.22% are 30–49 years old, and 14.76% are 50–64 years old, adding up to 97.72% of patients aged from 18 to 64 years old compared with 79.07% of cases categorized in the same age group for patients receiving hypoglycemic treatment. For exposure to canagliflozin, 99.82% of reports classified the targeted drug as the PS drug. The most common reporting source is consumers, representing 91.90% for canagliflozin-related cases associated with osteomyelitis. By contrast, approximately 50% of reports referring to hypoglycemic drugs are filed by consumers.

**TABLE 1:** Demographic of reports referring to blood glucose lowering drugs and osteomyelitis.

	**all blood glucose lowering drugs**			** **
	**(N = 486,927)**			
	**Osteomyelitis**			** **
	**(N = 2,888)**			
	**non-insulin**		**Insulin**	** **
	**(N = 2,634)**		**(N = 405)**	
	**sglt2i**	**non-sglt2i**	** **	** **
	**(N = 2,333)**	**(N = 484)**		
	**Canagliflozin**	** **	** **	** **
	**(N = 2,283)**			
**Gender:**				
Male	1678 (73.50%)	286 (59.09%)	233 (57.53%)	228600 (46.95%)
Female	605 (26.50%)	198 (40.91%)	172 (42.47%)	258080 (53.00%)
**Age:**				
0−9 yo	1 (0.04%)	0 (0.00%)	1 (0.25%)	2352 (0.48%)
10−17 yo	5 (0.22%)	2 (0.41%)	3 (0.74%)	8611 (1.77%)
18−29 yo	542 (23.74%)	66 (13.64%)	59 (14.57%)	63919 (13.13%)
30−49 yo	1352 (59.22%)	252 (52.07%)	193 (47.65%)	175913 (36.13%)
50−64 yo	337 (14.76%)	119 (24.59%)	102 (25.19%)	145152 (29.81%)
65−75 yo	44 (1.93%)	35 (7.23%)	36 (8.89%)	66503 (13.66%)
76−85 yo	2 (0.09%)	3 (0.62%)	1 (0.25%)	13499 (2.77%)
>85 yo	0 (0.00%)	0 (0.00%)	0 (0.00%)	3356 (0.69%)
**Yearly report:**				
2004	0 (0.00%)	5 (1.03%)	5 (1.23%)	3992 (0.82%)
2005	0 (0.00%)	5 (1.03%)	10 (2.47%)	5842 (1.20%)
2006	0 (0.00%)	11 (2.27%)	11 (2.72%)	15549 (3.19%)
2007	0 (0.00%)	3 (0.62%)	6 (1.48%)	16443 (3.38%)
2008	0 (0.00%)	5 (1.03%)	5 (1.23%)	11679 (2.40%)
2009	0 (0.00%)	15 (3.10%)	10 (2.47%)	13286 (2.73%)
2010	0 (0.00%)	10 (2.07%)	17 (4.20%)	16731 (3.44%)
2011	0 (0.00%)	17 (3.51%)	10 (2.47%)	21412 (4.40%)
2012	0 (0.00%)	19 (3.93%)	17 (4.20%)	19683 (4.04%)
2013	0 (0.00%)	12 (2.48%)	13 (3.21%)	21786 (4.47%)
2014	0 (0.00%)	19 (3.93%)	20 (4.94%)	31441 (6.46%)
2015	1 (0.04%)	28 (5.79%)	33 (8.15%)	59232 (12.16%)
2016	6 (0.26%)	23 (4.75%)	29 (7.16%)	41878 (8.60%)
2017	25 (1.10%)	37 (7.64%)	36 (8.89%)	38099 (7.82%)
2018	1402 (61.41%)	163 (33.68%)	86 (21.23%)	51442 (10.56%)
2019	635 (27.81%)	59 (12.19%)	42 (10.37%)	44095 (9.06%)
2020	207 (9.07%)	37 (7.64%)	35 (8.64%)	42959 (8.82%)
2021	7 (0.31%)	16 (3.31%)	20 (4.94%)	31373 (6.44%)
**Time to onset:**				
<1 day	16 (0.70%)	3 (0.62%)	5 (1.23%)	31500 (6.47%)
1−2 days	1 (0.04%)	1 (0.21%)	0 (0.00%)	4102 (0.84%)
2−3 days	3 (0.13%)	0 (0.00%)	1 (0.25%)	2295 (0.47%)
3−7 days	7 (0.31%)	1 (0.21%)	0 (0.00%)	5247 (1.08%)
1−2 weeks	3 (0.13%)	2 (0.41%)	2 (0.49%)	5775 (1.19%)
2−4 weeks	24 (1.05%)	1 (0.21%)	1 (0.25%)	7751 (1.59%)
1−3 months	59 (2.58%)	7 (1.45%)	4 (0.99%)	13061 (2.68%)
3−6 months	92 (4.03%)	5 (1.03%)	4 (0.99%)	9203 (1.89%)
0.5−1 year	130 (5.69%)	7 (1.45%)	1 (0.25%)	9962 (2.05%)
>1 year	255 (11.17%)	31 (6.40%)	22 (5.43%)	26770 (5.50%)
**Role of drug:**				
primary suspect	2279 (99.82%)	75 (15.50%)	129 (31.85%)	305376 (62.71%)
secondary suspect	223 (9.77%)	143 (29.55%)	91 (22.47%)	72440 (14.88%)
concomitant	3 (0.13%)	295 (60.95%)	248 (61.23%)	200501 (41.18%)
interacting	0 (0.00%)	0 (0.00%)	1 (0.25%)	1 (0.00%)
**Filed by:**				
consumer	2098 (91.90%)	252 (52.07%)	221 (54.57%)	242021 (49.70%)
other health-professional	110 (4.82%)	69 (14.26%)	47 (11.60%)	58259 (11.96%)
health professional	41 (1.80%)	19 (3.93%)	13 (3.21%)	16056 (3.30%)
physician	15 (0.66%)	103 (21.28%)	87 (21.48%)	114613 (23.54%)
lawyer	12 (0.53%)	4 (0.83%)	1 (0.25%)	5856 (1.20%)
pharmacist	7 (0.31%)	16 (3.31%)	9 (2.22%)	27861 (5.72%)
**Outcome:**				
hospitalization	1923 (84.23%)	372 (76.86%)	317 (78.27%)	194683 (39.98%)
disability	415 (18.18%)	67 (13.84%)	46 (11.36%)	11350 (2.33%)
death	18 (0.79%)	12 (2.48%)	25 (6.17%)	39769 (8.17%)
life-threatening	7 (0.31%)	15 (3.10%)	16 (3.95%)	24368 (5.00%)
required intervention	0 (0.00%)	3 (0.62%)	0 (0.00%)	2564 (0.53%)
congenital anomaly	0 (0.00%)	0 (0.00%)	0 (0.00%)	576 (0.12%)

N: case number; Osteomyelitis: reports referring to osteomyelitis as adverse event; insulin: insulin and its analogs; sglt2i: sodium-glucose co-transporter-2 inhibitors; Time to onset: timespan since the start date of therapy to the event date.

### 3.3 Disproportionality analyses and signal detection

**FIGURE 3:**
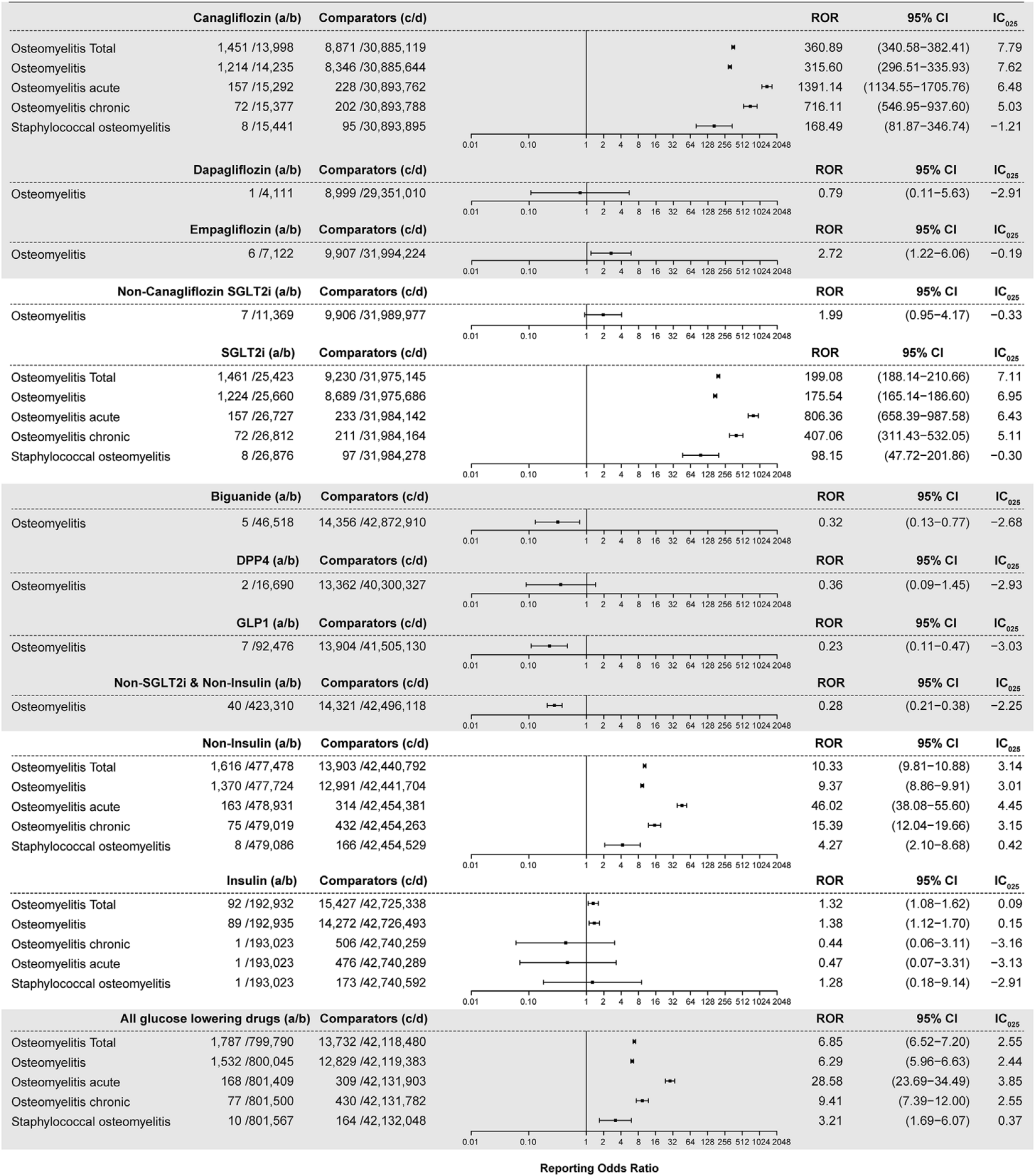
ROR and IC_025_ values of blood glucose-lowering drugs-associated osteomyelitis events, filtering diabetes as an indication. **(A)** Number of reports referring to both the targeted drug and the interested AE (targeted drug–osteomyelitis pair); **(B)** number of reports referring to the targeted drug paired with all reported AEs other than osteomyelitis; **(C)** number of reports referring to osteomyelitis concerning all drugs other than the targeted drug; **(D)** number of reports referring to all reported drug–AE pairs other than the targeted drug–osteomyelitis pair. IC_025_: lower limit of the information component of the Bayesian confidence propagation neural network.

**FIGURE 4:**
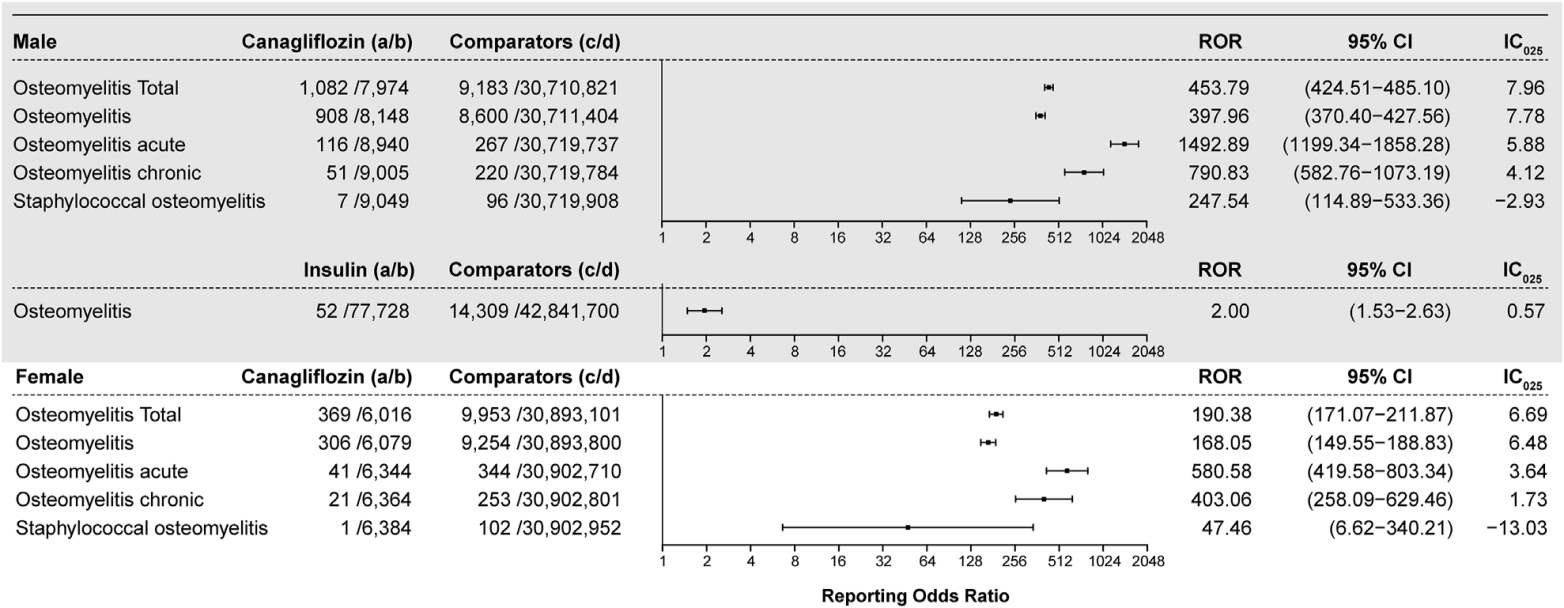
ROR and IC_025_ values of the blood glucose-lowering drug-associated osteomyelitis events, filtering gender. **(A)** Number of reports referring to both the targeted drug and the interested AE (targeted drug–osteomyelitis pair); **(B)** number of reports referring to the targeted drug paired with all reported AEs other than osteomyelitis; **(C)** number of reports referring to osteomyelitis concerning all drugs other than the targeted drug; **(D)** number of reports referring to all reported drug–AE pairs other than the targeted drug–osteomyelitis pair. IC_025_: lower limit of the information component of the Bayesian confidence propagation neural network; male: reports of male patients; female: reports of female patients.

All interested drug–osteomyelitis pairs were subjected to disproportionality analysis and BCPNN in duplicate, with filtering for the diabetes indication. Results are shown in Figure 3. In total, 1,451 osteomyelitis-related AEs were generated out of a total of 1,438 reports listing canagliflozin. The ROR value is 360.89 (95% CI 340.58–382.41) coupled with an IC_025_ value of 7.79. For each osteomyelitis-related AE individually, the number of reports with the canagliflozin–osteomyelitis pair was 1,214, generating an ROR of 315.60 (95% CI 296.51–335.93) and an IC_025_ of 7.62; the number or reports with the canagliflozin–osteomyelitis acute pair was 157, generating an ROR of 1,391.14 (95% CI 1134.55–1705.76) and an IC_025_ of 6.48; and the number of reports with the canagliflozin–osteomyelitis chronic pair was 72, generating an ROR of 716.11 (95% CI 546.95–937.60) and an IC_025_ of 5.03. All of the aforementioned signals are classed as strong signals. For the canagliflozin–staphylococcal osteomyelitis pair, a high ROR value was generated (168.49; 95% CI 81.87–346.74), but it was coupled with an IC_025_ of −1.21; therefore, it was cast out as a negative signal by BCPNN, as a false positive. For osteomyelitis associated with empagliflozin, the ROR value is 2.72 (95% CI 1.22–6.06) and the IC_025_ is −0.19 (Figure 3), whereas other SGLT2is as well as other hypoglycemic drugs, except for insulin, did not generate valid ROR values. Non-canagliflozin SGLT2is as a group could generate an ROR of 1.99 (95% CI 0.95–4.17) and an IC_025_ of −0.33, whereas other hypoglycemic drug groups including biguanides, DPP4, GLP1, and TZD did not generate valid ROR signals. Among all reports referring to osteomyelitis, 405 cases referred to insulin and its analogs and generated an ROR of 1.32 (95% CI 1.08–1.62) and an IC_025_ of 0.09, which could be considered as a weak signal, and 484 cases referred to non-insulin hypoglycemic drugs other than SGLT2is and could not generate valid ROR (0.28) and IC_025_ (−2.25) values, which meant no signal. With gender as a filter, a significant difference in the ROR of osteomyelitis associated with canagliflozin between male (ROR 453.79, 95% CI 424.51–485.10, IC_025_ 7.96) and female patients (ROR 190.38, 95% CI 171.07–211.87, IC_025_ 6.69) was observed. For the insulin–osteomyelitis pair, only male patients generated a weak signal (ROR 2.00, 95% CI 1.53–2.63, IC_025_ 0.57) (Figure 4).

### 3.4 Quarterly trend of ROR

**FIGURE 5:**
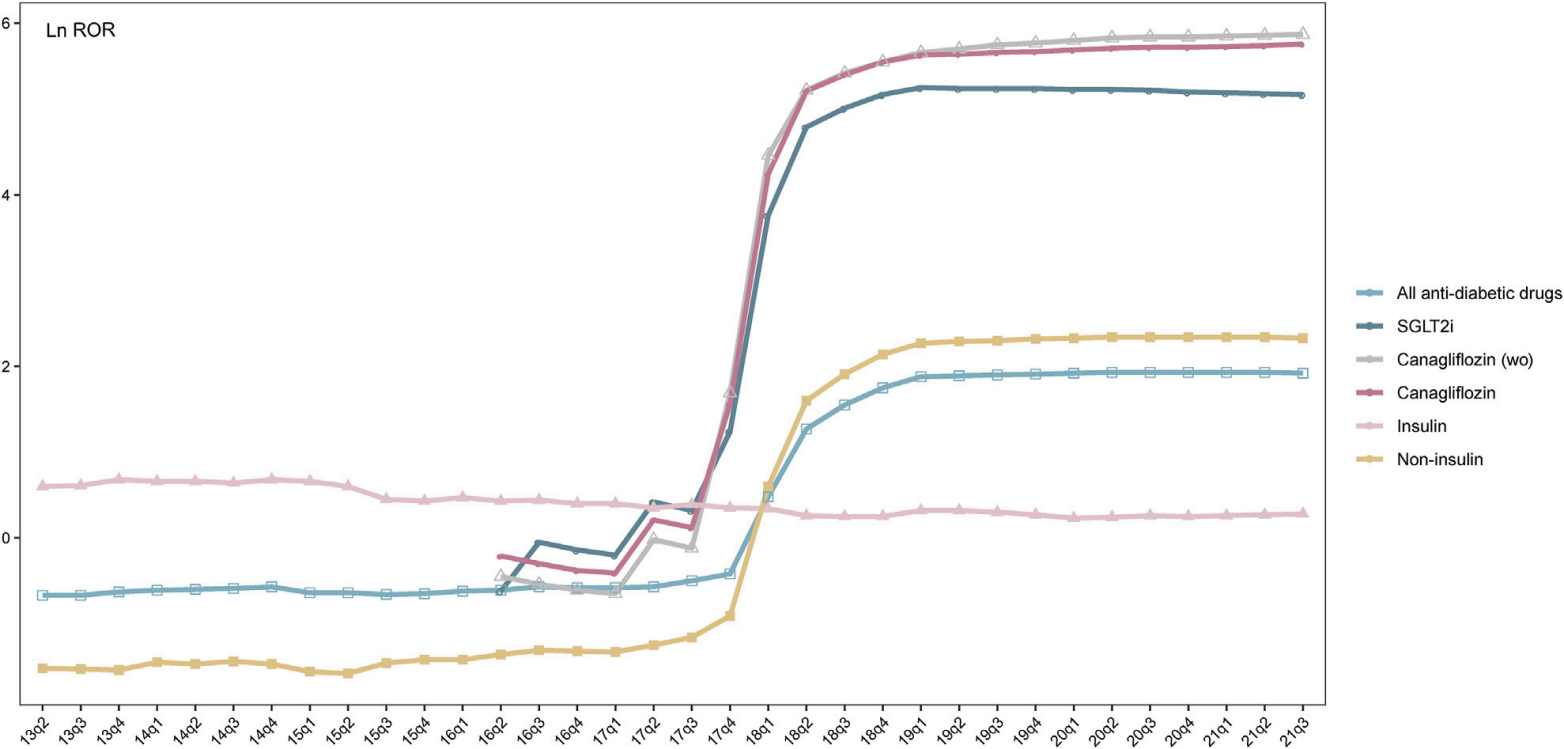
Trend of Ln ROR between all antidiabetic drugs, SGLT2is, insulin, non-insulin, canagliflozin (wo), canagliflozin, and osteomyelitis from the first quarter (Q1) up to the given quarter. *x*-axis: time in quarterly order; *y*-axis: Ln ROR; SGLT2is: sodium-glucose co-transporter-2 inhibitors; insulin: insulin and its analogs; non-insulin: antidiabetic drugs excluding insulin; canagliflozin (wo): canagliflozin without filtering diabetes as an indication.

To demonstrate the changing pattern of q-ROR (Supplementary Table S2 and Figure 5), the natural logarithm value of ROR (Ln ROR) was used as vertical coordinates and plotted against quarters of the year as horizontal coordinates. As shown in Figure 5, although reporting counts of the canagliflozin–osteomyelitis pair diminishes considerably with filtering diabetes as an indication compared with without the filtering, the curve of canagliflozin is almost overlapping with the curve of canagliflozin (wo), i.e., the curve without filtering diabetes as an indication. When hypoglycemic medication drugs excluding SGLT2is and insulin are investigated as the drug group, all the q-ROR values are below the ROR threshold value of 1 (Ln ROR 0), whereas the Ln ROR–time curve of insulin yielded a generally horizontal line, with the ROR value consistently within the range from 1 to 2 (Ln ROR range 0–1), since the second quarter (Q2) of 2013. As shown in Supplementary Table S2, during 18 years from 1 January 2004 to 30 September 2021, the q-ROR value of insulin is always above the recognition threshold of 1 and fluctuates consistently around a median of 1.57 (mean 1.71 ± 0.44 SD), and a valid ROR could be identified since the third quarter (Q3) of 2004. The curves of canagliflozin and SGLT2is start to generate valid ROR signals since as early as 2017 in the fourth quarter (Q4), whereas for any drug group including SGLT2is, the first valid ROR emerges in 2018 in Q1. For any drug group excluding canagliflozin, such as non-canagliflozin ATCA10 and non-SGLT2i ATCA10, no valid ROR signal is generated (Supplementary Table S2).

Chi2 tests were then applied to investigate the correlation between the series of q-RORs, using a null hypothesis claiming the prevalence of any two given series of q-RORs was the same. Among all series, canagliflozin, canagliflozin (wo), and SGLT2is shared the same pattern, although the scales of RORs were considerably different. Changing patterns of canagliflozin and SGLT2is demonstrate differences from insulin (*p* = 0.00) and other hypoglycemic drugs or drug groups (Supplementary Table S3). Another Chi2 test was introduced to determine the prevalence of q-ROR of the ATCA10–osteomyelitis pair before and after the approval of SGLT2is during the same time span, which is from Q2 of 2004 to Q4 of 2012 as serial A and from Q2 of 2013 to Q3 of 2021 as serial B. A *p*-value of 0.00924 was generated, and the null hypothesis was rejected.

## 4 Discussion

### 4.1 Panorama of AEs associated with hypoglycemic treatment

As shown in Figure 2, ketoacidosis, various infections, peripheral ischemia, renal impairment, and inflammation including osteomyelitis might be more likely to occur among SGLT2i users, especially for canagliflozin. Our findings suggest that SGLT2is increased the risk of these issues or were less effective on them. SGLT2i treatment for patients who suffered from ketoacidosis, cardiovascular issues, renal problems, and inflammation was, therefore, not recommended. Osteomyelitis and cellulitis are AEs unique to canagliflozin. Osteomyelitis is considered to greatly increase the risk of lower extremity amputation (Lavery et al., 2006; Game, 2010), and our findings indicated that exposure to canagliflozin could notably increase the risk of developing osteomyelitis, whereas other hypoglycemic drugs could reduce such risk. These events could be monitored as a critical warning before lower limb extremity amputation, especially due to osteomyelitis. By contrast, according to the FAERS data, hypoglycemic medications, except SGLT2is, showed encouraging curative effects on ketoacidosis, various infections, peripheral ischemia, renal impairment, and inflammation, which could be considered as complications of diabetes mellitus. Further studies should be undertaken to evaluate the risks *vs.* benefits of SGLT2is, and SGLT2is might not be recommended for patients who have suffered from such issues.

### 4.2 Osteomyelitis and canagliflozin

Most osteomyelitis-related cases were referred to canagliflozin, indicating that there might be a strong correlation between SGLT2i exposure, especially canagliflozin, and developing osteomyelitis according to the FAERS data. In this study, ROR and BCPNN methods were applied to investigate the association between hypoglycemic drugs and osteomyelitis. Signals with a high ROR value indicated strong disproportionality and a strong association between the targeted drug and AEs. Because the value of the ROR did not directly indicate the significance of a signal, all positive signals were validated by the BCPNN method. Strong signals associated with osteomyelitis were generated for canagliflozin or any drug groups containing canagliflozin, whereas weak signals were generated for insulin–osteomyelitis pairs, and no signal was generated for other hypoglycemic drugs or drug groups excluding canagliflozin and insulin. Therefore, these findings indicated that an association between canagliflozin treatment and osteomyelitis was convincing. The weak signal generated by the insulin–osteomyelitis pair might be explained by insulin exposure as well as the morbidity of diabetes since insulin treatment, indicating a proxy of disease severity or advanced disease stage (Davies et al., 2018; Pasquel et al., 2021), but the morbidity of diabetes might neither be a sufficient condition nor a necessary condition for a patient with diabetes to develop osteomyelitis. The total number of reports on targeted drugs presented notable differences with or without filtering diabetes as an indication, and the IC_025_ value of canagliflozin–osteomyelitis pairs with the filtering was lower than that without it, suggesting that excluding cases without a specific indication as diabetes resulted in diminishing the intensity of the BCPNN signal.

### 4.3 Gender differences

Among patients who developed osteomyelitis, 73.50% were male patients, whereas the gross gender ratio for each category of hypoglycemic drugs was relatively more balanced (Table 1). Moreover, disproportionality analysis was performed with gender as the filtering criterion, and the results suggested that there was a significant difference in the ROR of canagliflozin–osteomyelitis pairs between the two genders. Since filtering according to the aforementioned exclusion criteria had excluded all reports with infection known as a competing indication and reaction (Eckman et al., 1995), the gender ratio and differences in ROR and IC_025_ values between male and female patients were probably due to gender differences, and a negative correlation might have existed between glycosylated hemoglobin (HbA1c) and serum testosterone levels (Zhang et al., 2021). As displayed by the insulin–osteomyelitis pair, only the dataset of male patients could generate a valid ROR and a weak signal of BCPNN; thus, these findings support the hypothesis that male patients might be more likely to develop osteomyelitis. When exposure to the SGLT2i, canalization was the major factor for causing disproportionality, the dataset of male patients generated an ROR value three times higher than that of female patients. When the signals were validated by the BCPNN method, both genders generated strong signals (IC_025_ of 7.96 for male patients *vs*. IC_025_ of 6.69 for female patients), indicating that for reports of each gender, regardless of their differences in ROR values, canagliflozin presented a strong correlation with developing osteomyelitis (Figure 4).

### 4.4 Quarterly trend of ROR

A new approach, q-ROR, was introduced to demonstrate the developing trend of ROR, and the series of q-ROR values generated by different drugs or drug groups was subjected to Chi2 tests to determine their correlations statistically. Canagliflozin, SGLT2is, and ATCA10 demonstrated no prevalence difference, although there might be a gap in the scale of ROR values (Supplementary Table S2 and Figure 5), supporting the aforementioned speculation that the disproportionality of osteomyelitis-related reports was generated by canagliflozin. For hypoglycemic drugs other than canagliflozin and drug groups excluding SGLT2is, no positive signal was generated when paired with osteomyelitis-related AEs. These findings strongly indicated that the developing pattern of these drugs or drug groups was synchronized by the presence of canagliflozin. In a pharmacovigilance study, disproportionality emerges when a specific AE is associated with a given drug (Almenoff et al., 2007; Hou et al., 2014; Ang et al., 2016). In this study, we used q-ROR with the FAERS quarterly data and mimicked the accumulation of reports to the database in the real world. Starting from the setting date, a slice of data was added to the dataset in chronological order on a quarterly basis, and an ROR value from the setting date up to that quarter was calculated. A series of RORs was generated for any given interested drug**/**drug group–AE pair. Finally, the q-ROR value achieved equilibrium and approached its theoretical true value. For recently approved drugs with limited reports but with analogs that had been long approved, the q-ROR curve might be used to predict their association with interested AEs according to their precursors or as a drug group, such as ertugliflozin, luseogliflozin, remogliflozin, and other newly approved SGLT2is that fail to generate any positive signal. The q-ROR value of the insulin–osteomyelitis pair was always above the recognition threshold and fluctuated consistently around 1.5, and a positive signal could be identified since Q1 of 2005, whereas a series of q-RORs referring to any drug group excluding canagliflozin and insulin was below the threshold of 1 since 2005. Coupled with the dramatically increasing number of reports of canagliflozin-related osteomyelitis in the FAERS (25 cases in 2017 *vs*. 1,402 cases in 2018) (Table 1), the q-ROR pattern of canagliflozin ranges from 3.24 in Q4 of 2017 to 79.54 in Q4 of 2018 (Supplementary Table S2). This finding indicates that q-ROR could be used to monitor drug-induced ADRs unknown to premarketing trials as pharmacovigilance, when a dramatic rise in the ROR value is spotted for given drug–AE pairs and needs to be further verified by the BCPNN method.

### 4.5 Interfering caused by morbidities

Morbidity of diabetes mellitus is a risk factor for developing osteomyelitis, which occurs in approximately 10%–20% of patients with diabetes-related foot ulcers (Game, 2010), and osteomyelitis of the lower extremity is a commonly encountered problem in patients with diabetes (Butalia et al., 2008). In this study, such a dataset in the FAERS database was also equivalent to considering all hypoglycemic drugs as a drug group and filtering data with diabetes as an indication, which generated a signal considered to be caused by both the treatment and the morbidity. This dataset was also examined by the q-ROR method. The Chi2 test was used to compare a series of Ln ROR values before and after canagliflozin was approved in Q1 of 2013, and a *p*-value of 0.00924 (*p* < 0.05) indicated that a significant change in disproportionality of the diabetes–osteomyelitis combination, which was probably due to the exposure to SGLT2is, especially canagliflozin, because before the approval of SGLT2is, diabetes as a risk factor generated no positive signal when paired with osteomyelitis-related AEs.

Previous publications suggested that osteomyelitis of the lower extremity is a commonly encountered problem in patients with diabetes (Butalia et al., 2008) and occurred in approximately 10%–20% of patients with diabetes-related foot ulcers (Game, 2010). However, based on the FAERS data, drug or drug groups excluding canagliflozin and insulin generated no positive signal (Supplementary Table S2 and Figure 5) with osteomyelitis-related AEs. For insulin, the Ln ROR value was 1.71 ± 0.44, with a median of 1.57, before the approval of canagliflozin, and 1.53 ± 0.25, with a median of 1.45, since Q2 of 2013, when canagliflozin was approved as the first SGLT2i. This finding indicated that the morbidity of diabetes mellitus, even as a proxy of disease severity or advanced disease stage (Davies et al., 2018; Pasquel et al., 2021), might not be considered a significant interfering factor for drug-associated osteomyelitis based on the FAERS database. Therefore, this strengthened the results of the Chi2 tests between the q-ROR series that canagliflozin exposure might be the predominant cause of developing osteomyelitis for patients with diabetes, based on the FAERS database. By contrast, other widely used SGLT2is, such as dapagliflozin and empagliflozin, might not be associated with developing osteomyelitis. For recently approved SGLT2is that have not accumulated enough ADR reports for disproportionality analysis, predictions could be made based on the q-ROR pattern as pharmacovigilance on a quarterly basis.

### 4.6 Limitations

There are certain limitations that might undermine this study. Spontaneous reporting systems including the FAERS database were exposed to the biases inherent to pharmacovigilance studies. To the best of our knowledge, in 2018, Chang et al. (2018) mentioned the risk of osteomyelitis when discussing the association between SGLT2i treatment and lower extremity amputation among patients with T2D, and osteomyelitis of the lower extremity is a commonly encountered problem in patients with diabetes (Butalia et al., 2008) and occurs in approximately 10%–20% of patients with diabetes-related foot ulcers (Game, 2010). These publications coincidently matched with the outflow of osteomyelitis-related ADR reports and a surge in the ROR for the canagliflozin–osteomyelitis pair.

## 5 Conclusion

In conclusion, according to the FAERS data, most of the hypoglycemic agents demonstrated curative effects on preventing lower extremity amputation and osteomyelitis before such irreversible outcome, whereas SGLT2is were less effective on this issue. In this study, we investigated all hypoglycemic agents mapped in class A10 of the Anatomic Therapeutic Chemical Classification to provide insight into their association with referring AEs. Ketoacidosis, infection, peripheral ischemia, renal impairment, and inflammation might be more likely to occur among SGLT2i, especially canagliflozin, users. Osteomyelitis and cellulitis are AEs unique to canagliflozin and are, therefore, intensively discussed. ROR, IC_025_, and q-ROR tendencies of the canagliflozin–osteomyelitis pair were significantly different from those generated by the insulin–osteomyelitis pair, and there was no positive signal for hypoglycemic drugs paired with osteomyelitis other than canagliflozin and insulin. Our findings strongly indicated that canagliflozin treatment increases the risk of developing osteomyelitis from the very early stage of diabetes mellitus, before the advanced stage when insulin is prescribed. It is worth investigating whether SGLT2is can also result in the development of osteomyelitis in patients without diabetes, and the association between osteomyelitis and recently approved SGLT2is, when enough reports become available. Further studies are needed for a better understanding of the association between SGLT2i treatment and the risk of osteomyelitis.

## Data Availability

Publicly available datasets were analyzed in this study. These data can be found at: https://fis.fda.gov/extensions/FPD-QDE-FAERS/FPD-QDE-FAERS.html.
