# Supplementary material for: Sodium-glucose co-transporter-2 inhibitor (SGLT2i) treatment and risk of osteomyelitis: A pharmacovigilance study of the FAERS database
Source: Front Pharmacol. 2023 Feb 14;14:1110575. doi: 10.3389/fphar.2023.1110575 (PMC9971937; doi:10.3389/fphar.2023.1110575)
Supplement: Supplementary file 1 [file Table1.docx]

**Supplementary Table 1. Detailed IC_025_ of SGLT2i-AE pairs：**Lower limit of information component (IC_025_) SGLT2i, diabetes or insulin and risky AEs.

| **AE** | **Lower limit of information component (**IC_025_**)** | | | | | |
| --- | --- | --- | --- | --- | --- | --- |
|  | **diabetes** | **canagliflozin** | **dapagliflozin** | **empagliflozin** | **ertugliflozin** | **insulin** |
| acidosis | 1.07 | 2.91 | 2.25 | 3.29 | 0.00 | 1.54 |
| diabetic ketoacidosis | 3.57 | 6.48 | 6.47 | 6.62 | 0.75 | 3.43 |
| euglycaemic diabetic ketoacidosis | 4.00 | 5.87 | 6.83 | 7.76 | 0.00 | 3.16 |
| ketoacidosis | 3.57 | 5.43 | 6.60 | 6.99 | 0.00 | 3.34 |
| metabolic acidosis | 1.94 | 3.07 | 3.28 | 3.46 | 0.00 | 1.52 |
|  |  |  |  |  |  |  |
| acute kidney injury | 1.56 | 1.76 | 2.27 | 2.10 | 0.00 | 1.64 |
| creatinine renal clearance increased | 0.52 | 0.55 | 0.00 | 0.21 | 0.00 | 0.00 |
| renal abscess | 0.53 | 0.75 | 0.00 | 0.91 | 0.00 | 0.67 |
| renal impairment | 0.47 | 0.73 | 1.20 | 1.35 | 0.00 | 1.25 |
|  |  |  |  |  |  |  |
| renal pain | 0.46 | 1.17 | 0.00 | 0.00 | 0.00 | 0.00 |
| amputation | 2.75 | 3.00 | 0.00 | 0.91 | 0.00 | 1.46 |
| foot amputation | 3.24 | 4.04 | 0.89 | 1.32 | 0.00 | 1.44 |
| leg amputation | 2.76 | 4.29 | 0.48 | 2.01 | 0.00 | 1.06 |
| toe amputation | 3.38 | 5.35 | 2.83 | 3.22 | 0.00 | 1.14 |
|  |  |  |  |  |  |  |
| balanoposthitis | 2.49 | 2.89 | 3.42 | 3.97 | 0.00 | 1.23 |
| cellulitis | 0.56 | 2.16 | 0.34 | 0.64 | 0.00 | 0.93 |
| cholecystitis acute | 0.29 | 0.95 | 0.62 | 0.54 | 0.00 | 0.70 |
| gastroenteritis | 0.66 | 2.03 | 1.30 | 1.80 | 0.00 | 0.92 |
| necrotising fasciitis | 1.58 | 2.57 | 3.77 | 3.89 | 0.00 | 1.04 |
| osteomyelitis | 1.80 | 4.17 | 0.00 | 0.34 | 0.00 | 0.71 |
| osteomyelitis acute | 2.94 | 3.44 | 0.00 | 0.00 | 0.00 | 0.00 |
| osteomyelitis chronic | 1.82 | 1.88 | 0.00 | 0.00 | 0.00 | 0.00 |
| pancreatitis | 1.93 | 3.06 | 1.29 | 2.16 | 0.00 | 1.07 |
| prostatitis | 0.31 | 2.65 | 0.00 | 2.28 | 0.00 | 0.50 |
| pyelonephritis | 0.80 | 2.82 | 2.82 | 2.51 | 0.00 | 1.24 |
| pyelonephritis acute | 1.00 | 1.09 | 1.75 | 1.53 | 0.00 | 1.02 |
|  |  |  |  |  |  |  |
| diabetic foot infection | 3.46 | 5.04 | 0.00 | 0.82 | 0.00 | 0.88 |
| fungal infection | 1.06 | 4.20 | 2.71 | 3.15 | 0.00 | 0.00 |
| genital infection fungal | 2.89 | 5.01 | 0.04 | 2.02 | 0.00 | 0.00 |
| kidney infection | 0.05 | 0.52 | 0.00 | 0.00 | 0.00 | 0.00 |
| localised infection | 0.53 | 1.54 | 0.00 | 0.00 | 0.00 | 0.53 |
| urinary tract infection | 0.10 | 1.84 | 1.48 | 1.72 | 0.00 | 0.35 |
| urinary tract infection bacterial | 0.01 | 0.24 | 0.52 | 0.52 | 0.00 | 0.43 |
| vulvovaginal mycotic infection | 0.91 | 2.60 | 0.91 | 1.34 | 0.00 | 0.00 |
| vulvovaginal candidiasis | 0.55 | 1.89 | 1.16 | 1.48 | 0.00 | 0.06 |
| vulvovaginal pruritus | 0.85 | 1.09 | 2.24 | 1.61 | 0.00 | 0.00 |
|  |  |  |  |  |  |  |
| peripheral ischaemia | 0.39 | 2.23 | 0.86 | 1.61 | 0.00 | 1.62 |
| peripheral arterial occlusive disease | 1.11 | 1.15 | 1.19 | 1.00 | 0.00 | 1.65 |
| cerebral infarction | 0.84 | 2.49 | 2.68 | 1.93 | 0.00 | 0.78 |
| angina pectoris | 1.20 | 1.22 | 0.39 | 1.50 | 0.00 | 0.92 |
|  |  |  |  |  |  |  |
| fournier's gangrene | 3.75 | 4.79 | 5.19 | 6.76 | 0.00 | 2.72 |
| gangrene | 2.52 | 4.60 | 1.96 | 2.36 | 0.00 | 1.58 |
|  |  |  |  |  |  |  |
| anion gap increased | 1.75 | 2.72 | 0.00 | 2.77 | 0.00 | 1.50 |
| blood bicarbonate decreased | 0.74 | 2.39 | 1.50 | 1.55 | 0.00 | 1.15 |
| blood creatine increased | 0.16 | 0.87 | 1.40 | 1.90 | 0.00 | 1.11 |
| blood creatinine increased | 0.34 | 1.39 | 0.89 | 1.17 | 0.00 | 1.23 |
| blood lactic acid increased | 1.30 | 1.53 | 1.21 | 1.38 | 0.00 | 0.87 |
| blood ph decreased | 0.70 | 1.12 | 0.00 | 1.10 | 0.00 | 1.03 |
| blood potassium increased | 0.69 | 1.14 | 0.00 | 0.82 | 0.00 | 1.36 |
| blood triglycerides increased | 0.59 | 1.82 | 1.01 | 0.88 | 0.00 | 0.49 |
| blood urea increased | 0.34 | 1.26 | 0.00 | 1.65 | 0.00 | 1.43 |
| colon cancer | 0.24 | 1.05 | 1.00 | 0.00 | 0.00 | 0.00 |
| dehydration | 0.54 | 2.04 | 1.80 | 1.96 | 0.00 | 0.81 |
| diabetic foot | 3.26 | 4.12 | 0.97 | 2.25 | 0.00 | 2.31 |
| dizziness | 0.01 | 0.15 | 0.00 | 0.11 | 0.00 | 0.00 |
| drug prescribing error | 0.24 | 0.35 | 0.00 | 0.00 | 0.00 | 0.00 |
| extremity necrosis | 1.29 | 2.11 | 0.62 | 0.50 | 0.00 | 1.58 |
| gastrooesophageal reflux disease | 0.36 | 1.04 | 0.00 | 0.00 | 0.00 | 0.00 |
| genital rash | 0.75 | 1.37 | 0.00 | 2.40 | 0.00 | 0.00 |
| glomerular filtration rate decreased | 1.00 | 3.72 | 3.38 | 2.96 | 0.00 | 1.48 |
| glucose urine present | 1.96 | 2.16 | 3.22 | 2.47 | 0.00 | 1.58 |
| glycosuria | 1.43 | 2.03 | 2.48 | 3.46 | 0.00 | 1.69 |
| hepatic cirrhosis | 0.61 | 1.29 | 0.00 | 0.00 | 0.00 | 0.99 |
| hepatic steatosis | 0.77 | 1.01 | 0.79 | 0.77 | 0.00 | 0.86 |
| hyperkalaemia | 1.63 | 2.06 | 2.03 | 1.64 | 0.00 | 2.31 |
| hypernatraemia | 0.21 | 1.01 | 2.21 | 1.89 | 0.00 | 1.38 |
| hypertriglyceridaemia | 0.38 | 0.40 | 0.00 | 0.94 | 0.00 | 1.35 |
| hypophagia | 0.41 | 0.56 | 0.42 | 1.51 | 0.00 | 0.57 |
| hypovolaemia | 0.61 | 1.87 | 1.85 | 3.02 | 0.00 | 1.65 |
| ketonuria | 2.89 | 3.30 | 4.59 | 4.94 | 0.00 | 2.61 |
| ketosis | 3.29 | 3.44 | 5.00 | 4.70 | 0.00 | 3.02 |
| micturition disorder | 0.06 | 0.50 | 0.00 | 0.00 | 0.00 | 0.00 |
| necrosis | 0.04 | 1.47 | 0.30 | 1.23 | 0.00 | 0.91 |
| nephrolithiasis | 0.04 | 0.86 | 0.00 | 0.00 | 0.00 | 0.00 |
| nocturia | 0.08 | 0.78 | 0.78 | 0.69 | 0.00 | 0.00 |
| penile pain | 0.26 | 0.34 | 0.00 | 0.00 | 0.00 | 0.00 |
| pollakiuria | 0.85 | 1.94 | 1.87 | 2.79 | 0.00 | 0.00 |
| polycythaemia | 0.09 | 0.35 | 1.53 | 2.10 | 0.00 | 0.00 |
| polydipsia | 1.10 | 2.15 | 1.82 | 2.44 | 0.00 | 1.24 |
| polyuria | 1.26 | 2.94 | 3.41 | 3.45 | 0.00 | 1.31 |
| product use issue | 0.09 | 0.13 | 0.00 | 0.00 | 0.00 | 0.00 |
| rhabdomyolysis | 0.12 | 0.72 | 0.33 | 0.00 | 0.00 | 1.34 |
| sepsis | 0.02 | 1.03 | 0.98 | 1.24 | 0.00 | 1.11 |
| skin ulcer | 0.79 | 2.42 | 0.47 | 1.38 | 0.00 | 0.78 |
| thirst | 1.03 | 1.48 | 1.89 | 2.36 | 0.00 | 0.08 |
| treatment noncompliance | 0.07 | 1.58 | 0.00 | 0.00 | 0.00 | 0.61 |
| urine ketone body present | 2.59 | 4.95 | 3.74 | 5.01 | 0.00 | 1.92 |
| urine output increased | 1.49 | 3.30 | 1.21 | 1.45 | 0.00 | 0.00 |
| urosepsis | 0.80 | 2.59 | 2.89 | 3.37 | 0.00 | 1.36 |
| varices oesophageal | 0.33 | 1.18 | 0.00 | 0.23 | 0.00 | 0.99 |

AE: adverse event; Diabetes: cases with indication containing the key word “diabetes”; SGLT2i: sodium-glucose co-transporter-2 inhibitors; Insulin: insulin & its analogs; IC025: the lower limit of information component.
