# Supplementary material for: Sodium-glucose co-transporter-2 inhibitor (SGLT2i) treatment and risk of osteomyelitis: A pharmacovigilance study of the FAERS database
Source: Front Pharmacol. 2023 Feb 14;14:1110575. doi: 10.3389/fphar.2023.1110575 (PMC9971937; doi:10.3389/fphar.2023.1110575)
Supplement: Supplementary file 2 [file Table2.docx]

**Supplementary Table 2. Detailed quarterly ROR (q-ROR)：**ROR between all anti-diabetic drugs, SGLT2i, Insulin, Non-insulin, canagliflozin (wo), canagliflozin and osteomyelitis from the first quarter (q1) up to the given quarter**.**

|  | **All glucose lowering drugs** | | | | **SGLT2i** | | | | **canagliflozin (wo)** | | | | **canagliflozin** | | | | **Insulin** | | | | **Non-insulin** | | | |
| --- | --- | --- | --- | --- | --- | --- | --- | --- | --- | --- | --- | --- | --- | --- | --- | --- | --- | --- | --- | --- | --- | --- | --- | --- |
| **Q** | **Ln ROR** | **ROR** | **95%CI_025_** | **IC_025_** | **Ln ROR** | **ROR** | **95%CI_025_** | **IC_025_** | **Ln ROR** | **ROR** | **95%CI_025_** | **IC_025_** | **Ln ROR** | **ROR** | **95%CI_025_** | **IC_025_** | **Ln ROR** | **ROR** | **95%CI_025_** | **IC_025_** | **Ln ROR** | **ROR** | **95%CI_025_** | **IC_025_** |
| 04q1 | **0.27** | 1.31 | 0.18 | -2.91 |  | 0.00 | 0.00 | 0.00 |  | 0.00 | 0.00 | 0.00 |  | 0.00 | 0.00 | 0.00 | **1.30** | 3.68 | 0.51 | -3.68 |  | 0.00 | 0.00 | 0.00 |
| 04q2 | **-0.01** | 0.99 | 0.24 | -2.06 |  | 0.00 | 0.00 | 0.00 |  | 0.00 | 0.00 | 0.00 |  | 0.00 | 0.00 | 0.00 | **0.96** | 2.60 | 0.64 | -1.76 |  | 0.00 | 0.00 | 0.00 |
| 04q3 | **0.53** | 1.70 | 0.70 | -0.78 |  | 0.00 | 0.00 | 0.00 |  | 0.00 | 0.00 | 0.00 |  | 0.00 | 0.00 | 0.00 | **1.20** | 3.31 | 1.23 | -0.58 | **a10b** | 0.82 | 0.11 | -2.91 |
| 04q4 | **0.18** | 1.20 | 0.50 | -1.11 |  | 0.00 | 0.00 | 0.00 |  | 0.00 | 0.00 | 0.00 |  | 0.00 | 0.00 | 0.00 | **0.80** | 2.22 | 0.83 | -0.79 | **a10b** | 0.60 | 0.08 | -3.00 |
| 05q1 | **0.40** | 1.49 | 0.70 | -0.65 |  | 0.00 | 0.00 | 0.00 |  | 0.00 | 0.00 | 0.00 |  | 0.00 | 0.00 | 0.00 | **1.07** | 2.93 | 1.30 | -0.15 | **a10b** | 0.51 | 0.07 | -3.07 |
| 05q2 | **0.09** | 1.09 | 0.54 | -0.92 |  | 0.00 | 0.00 | 0.00 |  | 0.00 | 0.00 | 0.00 |  | 0.00 | 0.00 | 0.00 | **0.75** | 2.11 | 1.00 | -0.28 | **a10b** | 0.34 | 0.05 | -3.33 |
| 05q3 | **-0.09** | 0.91 | 0.46 | -1.13 |  | 0.00 | 0.00 | 0.00 |  | 0.00 | 0.00 | 0.00 |  | 0.00 | 0.00 | 0.00 | **0.59** | 1.81 | 0.86 | -0.44 | **a10b** | 0.28 | 0.04 | -3.49 |
| 05q4 | **-0.26** | 0.77 | 0.41 | -1.24 |  | 0.00 | 0.00 | 0.00 |  | 0.00 | 0.00 | 0.00 |  | 0.00 | 0.00 | 0.00 | **0.54** | 1.72 | 0.86 | -0.40 | **a10b** | 0.35 | 0.09 | -2.97 |
| 06q1 | **-0.41** | 0.67 | 0.37 | -1.40 |  | 0.00 | 0.00 | 0.00 |  | 0.00 | 0.00 | 0.00 |  | 0.00 | 0.00 | 0.00 | **0.57** | 1.76 | 0.91 | -0.30 | **a10b** | 0.25 | 0.06 | -3.37 |
| 06q2 | **-0.35** | 0.70 | 0.43 | -1.19 |  | 0.00 | 0.00 | 0.00 |  | 0.00 | 0.00 | 0.00 |  | 0.00 | 0.00 | 0.00 | **0.65** | 1.91 | 1.05 | -0.09 | **a10b** | 0.24 | 0.08 | -3.22 |
| 06q3 | **-0.30** | 0.74 | 0.49 | -1.02 |  | 0.00 | 0.00 | 0.00 |  | 0.00 | 0.00 | 0.00 |  | 0.00 | 0.00 | 0.00 | **0.81** | 2.26 | 1.36 | 0.28 | **a10b** | 0.24 | 0.09 | -3.14 |
| 06q4 | **-0.48** | 0.62 | 0.41 | -1.25 |  | 0.00 | 0.00 | 0.00 |  | 0.00 | 0.00 | 0.00 |  | 0.00 | 0.00 | 0.00 | **0.69** | 2.00 | 1.20 | 0.13 | **a10b** | 0.18 | 0.07 | -3.48 |
| 07q1 | **-0.57** | 0.57 | 0.38 | -1.36 |  | 0.00 | 0.00 | 0.00 |  | 0.00 | 0.00 | 0.00 |  | 0.00 | 0.00 | 0.00 | **0.70** | 2.02 | 1.24 | 0.18 | **a10b** | 0.16 | 0.06 | -3.70 |
| 07q2 | **-0.64** | 0.53 | 0.36 | -1.43 |  | 0.00 | 0.00 | 0.00 |  | 0.00 | 0.00 | 0.00 |  | 0.00 | 0.00 | 0.00 | **0.71** | 2.04 | 1.28 | 0.24 | **a10b** | 0.13 | 0.05 | -3.92 |
| 07q3 | **-0.75** | 0.47 | 0.32 | -1.59 |  | 0.00 | 0.00 | 0.00 |  | 0.00 | 0.00 | 0.00 |  | 0.00 | 0.00 | 0.00 | **0.66** | 1.94 | 1.22 | 0.18 | **a10b** | 0.12 | 0.04 | -4.10 |
| 07q4 | **-0.89** | 0.41 | 0.28 | -1.79 |  | 0.00 | 0.00 | 0.00 |  | 0.00 | 0.00 | 0.00 |  | 0.00 | 0.00 | 0.00 | **0.58** | 1.79 | 1.12 | 0.08 | **a10b** | 0.10 | 0.04 | -4.31 |
| 08q1 | **-0.95** | 0.39 | 0.27 | -1.85 |  | 0.00 | 0.00 | 0.00 |  | 0.00 | 0.00 | 0.00 |  | 0.00 | 0.00 | 0.00 | **0.54** | 1.71 | 1.08 | 0.02 | **a10b** | 0.11 | 0.05 | -4.08 |
| 08q2 | **-1.00** | 0.37 | 0.25 | -1.93 |  | 0.00 | 0.00 | 0.00 |  | 0.00 | 0.00 | 0.00 |  | 0.00 | 0.00 | 0.00 | **0.51** | 1.67 | 1.05 | -0.01 | **a10b** | 0.11 | 0.04 | -4.16 |
| 08q3 | **-1.07** | 0.34 | 0.24 | -2.02 |  | 0.00 | 0.00 | 0.00 |  | 0.00 | 0.00 | 0.00 |  | 0.00 | 0.00 | 0.00 | **0.48** | 1.62 | 1.02 | -0.05 | **a10b** | 0.10 | 0.04 | -4.27 |
| 08q4 | **-1.05** | 0.35 | 0.24 | -1.98 |  | 0.00 | 0.00 | 0.00 |  | 0.00 | 0.00 | 0.00 |  | 0.00 | 0.00 | 0.00 | **0.49** | 1.63 | 1.04 | -0.02 | **a10b** | 0.11 | 0.05 | -4.04 |
| 09q1 | **-1.02** | 0.36 | 0.25 | -1.92 |  | 0.00 | 0.00 | 0.00 |  | 0.00 | 0.00 | 0.00 |  | 0.00 | 0.00 | 0.00 | **0.45** | 1.57 | 1.00 | -0.07 | **a10b** | 0.14 | 0.07 | -3.60 |
| 09q2 | **-1.07** | 0.34 | 0.24 | -1.99 |  | 0.00 | 0.00 | 0.00 |  | 0.00 | 0.00 | 0.00 |  | 0.00 | 0.00 | 0.00 | **0.37** | 1.45 | 0.92 | -0.17 | **a10b** | 0.14 | 0.07 | -3.66 |
| 09q3 | **-1.10** | 0.33 | 0.23 | -2.04 |  | 0.00 | 0.00 | 0.00 |  | 0.00 | 0.00 | 0.00 |  | 0.00 | 0.00 | 0.00 | **0.33** | 1.39 | 0.89 | -0.22 | **a10b** | 0.13 | 0.07 | -3.72 |
| 09q4 | **-1.01** | 0.36 | 0.26 | -1.88 |  | 0.00 | 0.00 | 0.00 |  | 0.00 | 0.00 | 0.00 |  | 0.00 | 0.00 | 0.00 | **0.36** | 1.43 | 0.93 | -0.15 | **a10b** | 0.17 | 0.09 | -3.26 |
| 10q1 | **-0.97** | 0.38 | 0.28 | -1.80 |  | 0.00 | 0.00 | 0.00 |  | 0.00 | 0.00 | 0.00 |  | 0.00 | 0.00 | 0.00 | **0.44** | 1.55 | 1.04 | 0.00 | **a10b** | 0.17 | 0.09 | -3.30 |
| 10q2 | **-0.95** | 0.39 | 0.28 | -1.77 |  | 0.00 | 0.00 | 0.00 |  | 0.00 | 0.00 | 0.00 |  | 0.00 | 0.00 | 0.00 | **0.44** | 1.56 | 1.05 | 0.02 | **a10b** | 0.16 | 0.09 | -3.35 |
| 10q3 | **-0.91** | 0.40 | 0.30 | -1.70 |  | 0.00 | 0.00 | 0.00 |  | 0.00 | 0.00 | 0.00 |  | 0.00 | 0.00 | 0.00 | **0.43** | 1.54 | 1.04 | 0.01 | **a10b** | 0.17 | 0.10 | -3.23 |
| 10q4 | **-0.92** | 0.40 | 0.30 | -1.71 |  | 0.00 | 0.00 | 0.00 |  | 0.00 | 0.00 | 0.00 |  | 0.00 | 0.00 | 0.00 | **0.39** | 1.48 | 1.00 | -0.05 | **a10b** | 0.17 | 0.09 | -3.27 |
| 11q1 | **-0.89** | 0.41 | 0.31 | -1.66 |  | 0.00 | 0.00 | 0.00 |  | 0.00 | 0.00 | 0.00 |  | 0.00 | 0.00 | 0.00 | **0.41** | 1.51 | 1.03 | -0.01 | **a10b** | 0.16 | 0.09 | -3.30 |
| 11q2 | **-0.83** | 0.44 | 0.33 | -1.56 |  | 0.00 | 0.00 | 0.00 |  | 0.00 | 0.00 | 0.00 |  | 0.00 | 0.00 | 0.00 | **0.42** | 1.53 | 1.05 | 0.02 | **a10b** | 0.20 | 0.12 | -2.96 |
| 11q3 | **-0.82** | 0.44 | 0.34 | -1.54 |  | 0.00 | 0.00 | 0.00 |  | 0.00 | 0.00 | 0.00 |  | 0.00 | 0.00 | 0.00 | **0.40** | 1.49 | 1.02 | -0.01 | **a10b** | 0.20 | 0.12 | -2.94 |
| 11q4 | **-0.82** | 0.44 | 0.34 | -1.53 |  | 0.00 | 0.00 | 0.00 |  | 0.00 | 0.00 | 0.00 |  | 0.00 | 0.00 | 0.00 | **0.41** | 1.51 | 1.04 | 0.01 | **a10b** | 0.20 | 0.13 | -2.90 |
| 12q1 | **-0.77** | 0.46 | 0.36 | -1.46 |  | 0.00 | 0.00 | 0.00 |  | 0.00 | 0.00 | 0.00 |  | 0.00 | 0.00 | 0.00 | **0.43** | 1.54 | 1.07 | 0.05 | **a10b** | 0.22 | 0.14 | -2.76 |
| 12q2 | **-0.75** | 0.47 | 0.37 | -1.41 |  | 0.00 | 0.00 | 0.00 |  | 0.00 | 0.00 | 0.00 |  | 0.00 | 0.00 | 0.00 | **0.48** | 1.61 | 1.13 | 0.13 | **a10b** | 0.23 | 0.15 | -2.71 |
| 12q3 | **-0.72** | 0.49 | 0.38 | -1.37 |  | 0.00 | 0.00 | 0.00 |  | 0.00 | 0.00 | 0.00 |  | 0.00 | 0.00 | 0.00 | **0.53** | 1.70 | 1.21 | 0.23 | **a10b** | 0.22 | 0.14 | -2.72 |
| 12q4 | **-0.70** | 0.50 | 0.39 | -1.34 |  | 0.00 | 0.00 | 0.00 |  | 0.00 | 0.00 | 0.00 |  | 0.00 | 0.00 | 0.00 | **0.56** | 1.74 | 1.25 | 0.27 | **a10b** | 0.22 | 0.14 | -2.74 |
| 13q1 | **-0.69** | 0.50 | 0.40 | -1.31 |  | 0.00 | 0.00 | 0.00 |  | 0.00 | 0.00 | 0.00 |  | 0.00 | 0.00 | 0.00 | **0.59** | 1.80 | 1.29 | 0.32 | **a10b** | 0.22 | 0.14 | -2.74 |
| 13q2 | **-0.67** | 0.51 | 0.40 | -1.29 |  | 0.00 | 0.00 | 0.00 |  | 0.00 | 0.00 | 0.00 |  | 0.00 | 0.00 | 0.00 | **0.60** | 1.82 | 1.32 | 0.35 | **-1.52** | 0.22 | 0.14 | -2.76 |
| 13q3 | **-0.67** | 0.51 | 0.41 | -1.28 |  | 0.00 | 0.00 | 0.00 |  | 0.00 | 0.00 | 0.00 |  | 0.00 | 0.00 | 0.00 | **0.61** | 1.85 | 1.34 | 0.37 | **-1.53** | 0.22 | 0.14 | -2.77 |
| 13q4 | **-0.63** | 0.53 | 0.42 | -1.22 |  | 0.00 | 0.00 | 0.00 |  | 0.00 | 0.00 | 0.00 |  | 0.00 | 0.00 | 0.00 | **0.68** | 1.97 | 1.45 | 0.48 | **-1.54** | 0.21 | 0.14 | -2.78 |
| 14q1 | **-0.61** | 0.54 | 0.43 | -1.19 |  | 0.00 | 0.00 | 0.00 |  | 0.00 | 0.00 | 0.00 |  | 0.00 | 0.00 | 0.00 | **0.66** | 1.94 | 1.43 | 0.46 | **-1.45** | 0.23 | 0.15 | -2.63 |
| 14q2 | **-0.60** | 0.55 | 0.44 | -1.17 |  | 0.00 | 0.00 | 0.00 |  | 0.00 | 0.00 | 0.00 |  | 0.00 | 0.00 | 0.00 | **0.66** | 1.93 | 1.43 | 0.47 | **-1.47** | 0.23 | 0.15 | -2.66 |
| 14q3 | **-0.59** | 0.55 | 0.44 | -1.16 |  | 0.00 | 0.00 | 0.00 |  | 0.00 | 0.00 | 0.00 |  | 0.00 | 0.00 | 0.00 | **0.64** | 1.90 | 1.41 | 0.46 | **-1.44** | 0.24 | 0.16 | -2.61 |
| 14q4 | **-0.57** | 0.56 | 0.45 | -1.12 |  | 0.00 | 0.00 | 0.00 |  | 0.00 | 0.00 | 0.00 |  | 0.00 | 0.00 | 0.00 | **0.68** | 1.97 | 1.49 | 0.53 | **-1.47** | 0.23 | 0.15 | -2.64 |
| 15q1 | **-0.64** | 0.52 | 0.42 | -1.22 |  | 0.00 | 0.00 | 0.00 |  | 0.00 | 0.00 | 0.00 |  | 0.00 | 0.00 | 0.00 | **0.66** | 1.93 | 1.45 | 0.50 | **-1.56** | 0.21 | 0.14 | -2.77 |
| 15q2 | **-0.64** | 0.53 | 0.43 | -1.21 |  | 0.00 | 0.00 | 0.00 |  | 0.00 | 0.00 | 0.00 |  | 0.00 | 0.00 | 0.00 | **0.60** | 1.82 | 1.38 | 0.43 | **-1.58** | 0.21 | 0.14 | -2.80 |
| 15q3 | **-0.66** | 0.52 | 0.42 | -1.23 |  | 0.00 | 0.00 | 0.00 |  | 0.00 | 0.00 | 0.00 |  | 0.00 | 0.00 | 0.00 | **0.45** | 1.57 | 1.19 | 0.23 | **-1.46** | 0.23 | 0.16 | -2.60 |
| 15q4 | **-0.65** | 0.52 | 0.43 | -1.21 |  | 0.00 | 0.00 | 0.00 |  | 0.00 | 0.00 | 0.00 |  | 0.00 | 0.00 | 0.00 | **0.43** | 1.54 | 1.18 | 0.21 | **-1.42** | 0.24 | 0.17 | -2.54 |
| 16q1 | **-0.62** | 0.54 | 0.44 | -1.17 |  | 0.00 | 0.00 | 0.00 |  | 0.00 | 0.00 | 0.00 |  | 0.00 | 0.00 | 0.00 | **0.47** | 1.61 | 1.23 | 0.28 | **-1.42** | 0.24 | 0.17 | -2.54 |
| 16q2 | **-0.61** | 0.54 | 0.45 | -1.15 | **-0.62** | 0.54 | 0.08 | -3.05 | **-0.45** | 0.64 | 0.09 | -2.97 | **-0.21** | 0.81 | 0.11 | -2.91 | **0.43** | 1.53 | 1.18 | 0.21 | **-1.36** | 0.26 | 0.18 | -2.44 |
| 16q3 | **-0.57** | 0.57 | 0.47 | -1.08 | **-0.05** | 0.95 | 0.24 | -2.07 | **-0.54** | 0.58 | 0.08 | -3.01 | **-0.30** | 0.74 | 0.10 | -2.93 | **0.44** | 1.55 | 1.20 | 0.23 | **-1.31** | 0.27 | 0.19 | -2.35 |
| 16q4 | **-0.58** | 0.56 | 0.46 | -1.09 | **-0.14** | 0.87 | 0.22 | -2.13 | **-0.61** | 0.54 | 0.08 | -3.04 | **-0.38** | 0.69 | 0.10 | -2.95 | **0.40** | 1.50 | 1.16 | 0.18 | **-1.32** | 0.27 | 0.19 | -2.37 |
| 17q1 | **-0.58** | 0.56 | 0.46 | -1.10 | **-0.20** | 0.82 | 0.21 | -2.17 | **-0.65** | 0.52 | 0.07 | -3.06 | **-0.41** | 0.66 | 0.09 | -2.96 | **0.40** | 1.50 | 1.16 | 0.19 | **-1.33** | 0.26 | 0.19 | -2.38 |
| 17q2 | **-0.57** | 0.56 | 0.47 | -1.08 | **0.42** | 1.52 | 0.57 | -1.06 | **-0.02** | 0.98 | 0.24 | -2.06 | **0.21** | 1.24 | 0.31 | -1.92 | **0.35** | 1.42 | 1.10 | 0.12 | **-1.25** | 0.29 | 0.21 | -2.24 |
| 17q3 | **-0.50** | 0.60 | 0.51 | -0.97 | **0.32** | 1.38 | 0.52 | -1.14 | **-0.12** | 0.89 | 0.22 | -2.12 | **0.12** | 1.12 | 0.28 | -1.97 | **0.39** | 1.48 | 1.15 | 0.18 | **-1.16** | 0.31 | 0.23 | -2.09 |
| 17q4 | **-0.42** | 0.66 | 0.56 | -0.84 | **1.24** | 3.47 | 1.92 | 0.57 | **1.69** | 5.42 | 3.15 | 1.12 | **1.56** | 4.76 | 2.47 | 0.66 | **0.35** | 1.42 | 1.11 | 0.13 | **-0.91** | 0.40 | 0.30 | -1.69 |
| 18q1 | **0.48** | 1.62 | 1.46 | 0.52 | **3.76** | 43.12 | 36.96 | 4.82 | **4.46** | 86.79 | 76.68 | 5.77 | **4.24** | 69.40 | 59.36 | 5.30 | **0.34** | 1.40 | 1.10 | 0.11 | **0.60** | 1.82 | 1.60 | 0.66 |
| 18q2 | **1.27** | 3.57 | 3.32 | 1.65 | **4.79** | 120.45 | 110.48 | 6.39 | **5.22** | 184.41 | 170.42 | 6.92 | **5.21** | 183.57 | 168.17 | 6.84 | **0.26** | 1.30 | 1.02 | 0.01 | **1.60** | 4.96 | 4.58 | 2.11 |
| 18q3 | **1.55** | 4.70 | 4.41 | 2.03 | **5.01** | 149.77 | 139.22 | 6.72 | **5.42** | 225.17 | 210.60 | 7.22 | **5.40** | 221.23 | 205.43 | 7.15 | **0.25** | 1.28 | 1.01 | 0.00 | **1.91** | 6.74 | 6.30 | 2.54 |
| 18q4 | **1.75** | 5.78 | 5.46 | 2.31 | **5.17** | 175.50 | 164.50 | 6.95 | **5.55** | 258.07 | 243.01 | 7.41 | **5.55** | 256.91 | 240.59 | 7.38 | **0.25** | 1.29 | 1.02 | 0.02 | **2.14** | 8.47 | 7.97 | 2.85 |
| 19q1 | **1.88** | 6.55 | 6.21 | 2.47 | **5.25** | 190.44 | 179.33 | 7.06 | **5.66** | 288.40 | 272.92 | 7.56 | **5.63** | 278.86 | 262.35 | 7.50 | **0.32** | 1.37 | 1.11 | 0.13 | **2.27** | 9.71 | 9.18 | 3.04 |
| 19q2 | **1.89** | 6.62 | 6.28 | 2.49 | **5.24** | 188.70 | 177.83 | 7.06 | **5.70** | 299.17 | 283.59 | 7.61 | **5.64** | 281.76 | 265.27 | 7.52 | **0.32** | 1.38 | 1.11 | 0.14 | **2.29** | 9.84 | 9.31 | 3.06 |
| 19q3 | **1.90** | 6.69 | 6.35 | 2.50 | **5.24** | 188.33 | 177.65 | 7.06 | **5.75** | 313.35 | 297.61 | 7.68 | **5.66** | 285.89 | 269.42 | 7.54 | **0.30** | 1.35 | 1.09 | 0.11 | **2.30** | 9.98 | 9.46 | 3.08 |
| 19q4 | **1.91** | 6.75 | 6.42 | 2.52 | **5.24** | 188.79 | 178.23 | 7.07 | **5.77** | 320.28 | 304.45 | 7.71 | **5.67** | 291.25 | 274.68 | 7.57 | **0.27** | 1.31 | 1.06 | 0.07 | **2.32** | 10.16 | 9.63 | 3.10 |
| 20q1 | **1.92** | 6.83 | 6.49 | 2.53 | **5.23** | 187.42 | 177.08 | 7.07 | **5.80** | 331.54 | 315.52 | 7.75 | **5.69** | 296.48 | 279.82 | 7.60 | **0.23** | 1.26 | 1.02 | 0.02 | **2.33** | 10.28 | 9.75 | 3.12 |
| 20q2 | **1.93** | 6.89 | 6.55 | 2.55 | **5.23** | 187.37 | 177.13 | 7.07 | **5.83** | 339.07 | 322.91 | 7.78 | **5.71** | 301.04 | 284.29 | 7.62 | **0.24** | 1.27 | 1.02 | 0.02 | **2.34** | 10.38 | 9.85 | 3.14 |
| 20q3 | **1.93** | 6.92 | 6.58 | 2.56 | **5.22** | 185.40 | 175.34 | 7.06 | **5.84** | 342.54 | 326.35 | 7.80 | **5.72** | 303.85 | 287.06 | 7.64 | **0.26** | 1.29 | 1.05 | 0.05 | **2.34** | 10.41 | 9.89 | 3.14 |
| 20q4 | **1.93** | 6.88 | 6.55 | 2.55 | **5.20** | 182.16 | 172.31 | 7.05 | **5.84** | 344.29 | 328.05 | 7.81 | **5.72** | 306.10 | 289.21 | 7.65 | **0.25** | 1.28 | 1.04 | 0.04 | **2.34** | 10.38 | 9.86 | 3.14 |
| 21q1 | **1.93** | 6.86 | 6.53 | 2.55 | **5.19** | 179.54 | 169.84 | 7.03 | **5.85** | 346.48 | 330.17 | 7.82 | **5.73** | 308.90 | 291.88 | 7.66 | **0.26** | 1.30 | 1.06 | 0.07 | **2.34** | 10.33 | 9.81 | 3.14 |
| 21q2 | **1.93** | 6.86 | 6.53 | 2.55 | **5.18** | 177.33 | 167.78 | 7.02 | **5.86** | 350.06 | 333.60 | 7.84 | **5.74** | 312.48 | 295.29 | 7.68 | **0.27** | 1.31 | 1.07 | 0.08 | **2.34** | 10.34 | 9.82 | 3.14 |
| 21q3 | **1.92** | 6.85 | 6.52 | 2.55 | **5.17** | 175.33 | 165.91 | 7.01 | **5.87** | 353.85 | 337.24 | 7.86 | **5.76** | 316.23 | 298.85 | 7.70 | **0.28** | 1.32 | 1.08 | 0.09 | **2.33** | 10.33 | 9.81 | 3.14 |

Q: quarter; Canagliflozin (wo): canagliflozin without filtering diabetes as indication; SGLT2i: sodium-glucose co-transporter-2 inhibitors; Insulin: insulin & its analogs; Ln ROR: natural algorithm value of ROR; 95%CI025: the lower limit of 95% confidence interval; IC025: the lower limit of information component.
