# Supplementary material for: Sodium-glucose co-transporter-2 inhibitor (SGLT2i) treatment and risk of osteomyelitis: A pharmacovigilance study of the FAERS database
Source: Front Pharmacol. 2023 Feb 14;14:1110575. doi: 10.3389/fphar.2023.1110575 (PMC9971937; doi:10.3389/fphar.2023.1110575)
Supplement: Supplementary file 3 [file Table3.docx]

**Supplement table 3. Cross table of chi square test** **with null hypothesis claiming the prevalence of any two-given series of q-ROR was the same.** A10: all glucose lowering drugs; Canagliflozin (wo): canagliflozin without filtering diabetes as indication; SGLT2i: sodium-glucose co-transporter-2 inhibitors; Insulin: insulin & its analogs.

| **P value** | **A10** | **Insulin** | **Non-insulin** | **SGLT2i** | **Canagliflozin** | **Canagliflozin (wo)** |
| --- | --- | --- | --- | --- | --- | --- |
| **A10** | **-** | **0.15** | **1.00** | **0.00** | **0.00** | **0.00** |
| **Insulin** | **0.15** | **-** | **0.00** | **0.00** | **0.00** | **0.00** |
| **Non-insulin** | **1.00** | **0.00** | **-** | **0.00** | **0.00** | **0.00** |
| **SGLT2i** | **0.00** | **0.00** | **0.00** | **-** | **0.99** | **0.53** |
| **Canagliflozin** | **0.00** | **0.00** | **0.00** | **0.99** | **-** | **1.00** |
| **Canagliflozin (wo)** | **0.00** | **0.00** | **0.00** | **0.53** | **1.00** | **-** |
